# Effect of embryo morphology and morphometrics on implantation of vitrified day 3 embryos after warming: a retrospective cohort study

**DOI:** 10.1186/s12958-016-0175-8

**Published:** 2016-07-30

**Authors:** Elia Fernandez Gallardo, Carl Spiessens, Thomas D’Hooghe, Sophie Debrock

**Affiliations:** University Hospitals Leuven, Leuven University Fertility Center, KU Leuven—University of Leuven, Herestraat 49, Leuven, B-3000 Belgium

**Keywords:** Vitrified/warmed embryo, Embryo morphology, Morphometrics, Frozen embryo transfer, Implantation rate

## Abstract

**Background:**

Characteristics routinely used to evaluate embryo quality after thawing include number of blastomeres survived and presence of mitosis resumption after overnight culture. It is unknown to which extent symmetry and fragmentation affect implantation after warming and whether application of stricter criteria either before vitrification or after warming would improve implantation rate (IR) of vitrified/warmed embryos. This study aimed to find new parameters to improve selection criteria for vitrification and for transfer after warming.

**Methods:**

Firstly, we evaluated standard morphological characteristics (intact survival, mitosis resumption, number of blastomeres, symmetry and fragmentation) of 986 warmed day 3 embryos and, from a subset of 654, we evaluated morphometric characteristics (fragmentation, symmetry and volume change). Secondly, we tested the hypothesis that IR of day 3 vitrified/warmed embryos is influenced by morphometric characteristics. IR per embryo transferred was calculated using embryos that were transferred in a single embryo transfer (SET) or a double embryo transfer (DET) with either 0 or 100 % implantation (830/986). We investigated the significant differences in IR between the different categories of a specific characteristic. These categories were based on our standard embryo evaluation system. The statistical tests Chi-square, Fisher’s exact or Cochrane-Armitage were used according to the type and/or categories of the variable.

**Results:**

The 986 embryos were transferred in 671 FET cycles with 16.9 % (167/986) IR. After exclusion of DET with 1 embryo implanted, IR per embryo transferred was 12.4 % (103/830). Embryo symmetry, fragmentation and volume change in vitrified/warmed day 3 embryos were not associated with IR. However, when mitosis resumption was present after overnight culture, intact embryos reached significantly higher IR than non-intact embryos and only when the embryo compacted after overnight culture the number of cells damaged after warming had no effect on IR. Concretely, embryos with 8 cells after warming or >9 cells after overnight culture–including compacted embryos–reached the highest IR (>15 %) while embryos with <6 cells after warming or with ≤6 cells after overnight culture had extremely low IR (<1 %).

**Conclusions:**

IR of vitrified embryos is determined by the number of cells lost, by the occurrence of mitosis resumption, and by the specific number of blastomeres present but not by fragmentation, blastomere symmetry or volume change. Unselecting embryos for cryopreservation because of fragmentation >10 % and/or symmetry < 75 % only leads to unwanted loss of embryos with acceptable implantation potential.

**Trial registration:**

Retrospectively registered NCT02639715.

## Background

Embryo cryopreservation is an essential part of treatment with Assisted Reproductive Technology (ART). Two embryo cryopreservation methods are commonly used: slow freezing and vitrification. Vitrification has progressively substituted slow freezing in the in vitro fertilization (IVF) lab routine due to the reported significantly higher survival and intact survival rates [1–3], and due to the subsequent higher implantation, pregnancy [3] and live birth rates per embryo warmed [4].

In current practice, supernumerary embryos of sufficient morphological quality resulting from ART are cryopreserved at either cleavage stage (day 2 or day 3) or at blastocyst stage (day 5 or day 6). While culture until blastocyst stage might select against less viable embryos leading to increased pregnancy rate per transfer, freezing at blastocyst stage decreases significantly the number of embryos frozen per cycle, reducing significantly the cumulative pregnancy rate when compared with freezing at cleavage stage [5]. At the moment, there is not sufficient evidence to choose freezing embryos at blastocyst stage rather than on cleavage stage. For that reason we focused our study on day 3 cleavage stage embryos.

Characteristics that are routinely used to evaluate quality of cleavage stage embryos after thawing include the number of blastomeres survived and the presence of mitosis resumption after overnight culture. The relation between these characteristics and implantation potential has been the focus of several studies, mainly performed with slow frozen embryos [6–9]. In embryos slow frozen/thawed on day 3, implantation rate (IR) has been reported to decrease significantly when the number of blastomeres survived is lower than 25 % [6], or when mitosis is not resumed during 20–24 h overnight culture after thawing [8–10]. However, IR is not affected in 7–8 cell slow frozen/thawed embryos when 2 or less cells are damaged [7], or in vitrified/warmed day 3 embryos with up to 2 cells damaged, compared to embryos with no cells damaged, as long as the embryo continues to cleave after overnight culture [10].

In the Alpha consensus paper on key performance indicators related to cryopreservation, it was reported that number of cells, embryo symmetry and fragmentation were similar before vitrification and after warming, but no data were reported on the effect of embryo symmetry and fragmentation after vitrification/warming on implantation rate [11]. During fresh embryo culture, it is known that cleavage stage embryos with asymmetrical blastomeres and/or with fragmentation >10 % are considered to have an impaired implantation potential compared to embryos with symmetrical blastomeres and/or with <10 % fragmentation, [12]. The degree of fragmentation during fresh culture has been related to the incidence of aneuploidies [13, 14] and the asymmetric embryo cleavage is associated with a lower pregnancy rate and higher degree of chromosomal aberration [13, 15, 16]. For that reason, and in order to improve the cost/effectiveness of a fresh IVF cycle and subsequent frozen-thawed embryo transfer (FET) cycles, embryo selection is done before cryopreservation based on morphological characteristics. In the Alpha consensus meeting on cryopreservation, it has been recommended that only optimal cleavage stage embryos should be cryopreserved [12], i.e. 4-cell stage embryos at 44 ± 1 h post insemination, 8-cell stage embryos at 68 ± 1 h post insemination with <10 % fragmentation, stage-specific cell size and no multinucleation. However, in practice, most authors report less strict criteria for embryo selection before cryopreservation [4, 10, 17–27]. At present, it is not clear if the stricter embryo selection criteria for cryopreservation, reported in the Alpha consensus, actually result in higher IR compared to clinical practices where less strict criteria are applied. This is clinically important, since it is not in the interest of the patient to discard embryos with sufficient quality and sufficient survival and implantation potential after thawing/warming, even if they do not meet the Alpha consensus criteria. In order to resolve this issue, it is important to determine to which extent implantation rate per embryo after vitrification/warming is determined by not only morphological (i.e. number of blastomeres, symmetry and fragmentation, intact survival and mitosis resumption) but also morphometric (i.e., total cell volume, symmetry, fragmentation and volume change) characteristics. Indeed, in previous research, we demonstrated in fresh cycles that total embryo volume is associated with pregnancy in a quadratic nature, i.e. both lower and higher volumes were associated with a lower pregnancy rate, based on a computer assisted embryo scoring system calculating both embryo symmetry and fragmentation from individual blastomere volume and total cell volume (TCV) of the embryo [28]. Moreover, the morphometric analysis has the advantages of reducing the intra- and inter-observer variability, reducing subjectivity and allowing evaluation without time restriction [29].

In order to find new morphometric embryo parameters that can improve the selection criteria for both vitrification and embryo transfer after warming, we tested the hypothesis that the implantation rate of embryos warmed after vitrification on day 3 is influenced by morphometric characteristics (i.e., total cell volume, symmetry, fragmentation and volume change). In addition we also studied IR related to standard embryo morphological characteristics after warming (i.e. number of blastomeres, symmetry and fragmentation, intact survival and mitosis resumption).

## Methods

### Patient and embryo selection

In this retrospective analysis, the study population was selected from embryos warmed between September 2011 and December 2014 in our center. All survived and transferred embryos from patients with female age < 40 years at oocyte retrieval (OR) were included. Patients with female age >40 years at OR, cycles with preimplantation genetic diagnosis and donated gametes were excluded. The study population consisted, in total, of 986 embryos from 424 patients transferred in 671 FET cycles.

All procedures were performed according to the Helsinki declaration on Human Experimentation. The study was approved by the Commission for Medical Ethics of the university Hospital Leuven (approval reference number S55685) and registered as a clinical trial (NCT02639715).

### Fresh cycle, embryo evaluation and cryopreservation by vitrification

In fresh cycles, ovarian stimulation and luteal supplementation were performed as described previously [30]. Oocyte aspiration, insemination and embryo culture were carried out as foresaid [4]. Embryos were evaluated for fertilization on day 1 after OR (16–20 h after insemination/injection), and for quality on day 2 (41–44 h after insemination/injection) and day 3 (66–71 h after insemination/injection). Embryo quality was assessed using the manual scoring system of the Leuven University Fertility Centre which is based on the visual evaluation of the number and size of blastomeres and the degree of fragmentation [29]. Briefly, embryos were assigned to one of the 4 categories according to their degree of fragmentation (0 %, 1–10 %, 10–25 %, >25 %) and to one of the 3 categories according to their degree of blastomere symmetry (>75 %, 75–50 %, <50 %). On day 3 one or two embryos were chosen for transfer –according to the Belgian law [31]– based on the manual scoring system.

Supernumerary embryos were cryopreserved on day 3 after OR if the quality was sufficient. This was defined as embryos in ≥ 6 cell stage on day 3 with ≤ 25 % fragmentation and with symmetry > 50 % on day 3 [4]. Embryos were vitrified using Vit Kit®–Freeze (Irvine Scientific, Newtownmountkennedy, Ireland), loaded into CBS-VIT-High Security straws (CBS, Cryo Bio System, L’Aigle, France) and plunged directly into liquid nitrogen. Embryos were further stored in vapour phase nitrogen container.

### FET cycles

Warmed embryos were transferred in natural cycles, stimulated cycles (gonadotrophin or clomiphene citrate) or hormonal replacement cycles as described previously [32]. Straws were warmed following manufacturer’s protocol Vit Kit®-Thaw (Irvine Scientific, Newtownmountkennedy, Ireland) until the number of survived embryos was equal to the number of requested embryos for transfer. A maximum of two embryos were replaced as determined by Belgian law [33].

After warming, embryos were cultured overnight in GM501 medium (Gynemed, Lensahn, Germany) under mineral oil (Gynemed) at 37 °C, pH 7.25–7.35 in a standard incubator (Sanyo MCO-20AIC, Osaka, Japan).

### Manual morphological evaluation after warming

Embryo quality was evaluated in all embryos (*n* = 986) immediately after warming (5–30 min) to evaluate survival and after overnight culture (20–24 h) to evaluate mitosis resumption. Fragmentation and symmetry were manually scored at both times. Embryos were considered survived if they had ≥ 50 % of cells intact immediately after warming and were considered intact survived if 100 % of the blastomeres had survived. Mitosis resumption after overnight culture was defined as an increase of the number of blastomeres or compaction of the survived embryos after overnight culture. No selection was performed in the warmed embryos for transfer. Thus, all embryos with or without mitosis resumption were transferred.

### Computer based morphometric analysis of embryos before and after warming and overnight culture

On a subset of embryos warmed between January and December 2014 (*n* = 654) multilevel images composed of 40 different focal planes were taken on day 1 after OR, at freezing (day 3), after warming and after overnight culture. These pictures were only available in this subset, since multilevel imaging was not performed in our laboratory before January 2014 or after December 2014. The available images were analysed retrospectively using Fertimorph Software (CellCura Software Solutions Copenhagen, Denmark). Using this software the total cell volume (TCV) of the embryo was calculated based on the manual drawing of the two diameters of every blastomere [29]. Criteria were pre-established to differentiate a blastomere and a fragment based on the findings by Hnida et al. [34] and Johanson et al. [35]. A blastomere should be ≥40 μm on day 3 when the embryo had ≤8 blastomeres. When the embryo had >8 blastomeres the minimum diameter for a blastomere was established as 35 μm. Missing values were present when no measurements could be performed, i.e. if the embryo was compacted or if the image was not stored.

Subsequently, the TCV of the embryo was used to calculate four parameters: fragmentation on day of freezing, blastomere symmetry, and volume change. To calculate the fragmentation (%) it was assumed that TCV stays constant through the embryo development. It was calculated as shown in Equation 1. Fragmentation results in negative value when the TCV_day3_ > TCV_day1_. In that case fragmentation was considered 0 %. If fragmentation resulted in ≤ -20 % the embryos were excluded from the analysis.

Equation 1. Calculation of fragmentation on day 3 based on Total cell volume (TCV).

$$ Fragmentation\ \left(\%\right)=\frac{\left(TC{V}_{Day1}-TC{V}_{Day3}\right)}{TC{V}_{Day1}}\cdot 100 $$

Symmetry (%) was calculated as shown in Equation 2. More symmetrical blastomeres result in a higher %.

Equation 2. Calculation of blastomere symmetry in embryos.

$$ Symmetry\ \left(\%\right)=100-\left(\frac{Diameter_{Biggest\  blastomere}-{Diameter}_{Smallest\  blastomere}}{Diameter_{Biggest\  blastomere}},\cdot, 100\right. $$

Since cryopreservation media cause exchange of water and medium through the embryo membrane, the difference in volume between day 3 and day of warming and the difference in volume between the day of warming and after overnight culture were also of interest.

Fragmentation using morphometrics was only measured before embryo vitrification since the measurement assumes that the total embryo volume does not vary during the days of development. After vitrifying and warming water is substituted by cryoprotectant and vice versa, which causes successive phases of shrinkage and re-expansion [11] impeding to assume that the embryo volume is constant after warming. Instead of fragmentation, the volume change after warming and after overnight culture was calculated using morphometrics.

### Study design and statistical analysis

First, morphological characteristics were described from a large dataset of warmed and survived embryos (*n* = 986) and then, from a subset of 654 embryos, morphometric characteristics were measured. Moreover, each morphological (Table 1) and morphometric (Table 3) characteristic was divided in different categories based on the categories in the standard scoring system used in our clinic and IR was calculated for each of the categories. IR per embryo transferred was defined as the presence of intrauterine sac with fetal heartbeat at 6–8 weeks after embryo transfer and could only be calculated for embryos that were transferred in a single embryo transfer (SET) or a double embryo transfer (DET) with either 0 or 100 % implantation. When the variable had 2 categories, independency between the variable and implantation was tested by Chi-square. When the compared categories were unbalanced and when at least one group had 5 or less counts Fisher’s exact test was used instead. When the variables had more than 2 ordinal categories, Cochran-Armitage trend test was used to detect their relation with IR. All statistical tests were performed in R with α<0.05 and graphs were plotted using excel.

**Table 1:** Morphological characteristics of vitrified/warmed embryos

	Category	After warming	After overnight culture
		n (%)	n (%)
N blastomeres^a^	<6	69 (7.0)	26 (2.6)
	6	114 (11.5)	35 (3.5)
	7	206 (20.9)	61 (6.2)
	8	315 (32.0)	105 (10.6)
	9	133 (13.5)	99 (10.0)
	>9	149 (15.1)	356 (36.1)
	M/EB	-	304 (30.8)
Fragmentation^b^	0 %	306 (31.0)	165 (16.7)
	1–10 %	494 (50.1)	356 (36.1)
	10–25 %	175 (17.7)	148 (15.0)
	>25 %	6 (0.6)	13 (1.3)
	M/EB	4 (0.4)	304 (30.8)
Symmetry^b^	>75 %	224 (22.7)	76 (7.7)
	75–50 %	558 (56.6)	365 (37.1)
	<50 %	200 (20.3)	241 (24.4)
	M/EB	4 (0.4)	304 (30.8)

Number of blastomeres, degree of fragmentation and symmetry were based on the manual scoring system

^a^Embryos that were in morula (M) stage at freezing and after warming (*n* = 4) are included in the group of >9 blastomeres

^b^Symmetry and fragmentation for embryos in morula (M) or early blastocyst (EB) was not evaluated

## Results

### Morphological characteristics

The proportion of embryos observed in the different categories of number of blastomeres, symmetry and fragmentation are summarized in Table 1.

**Fig. 1:**
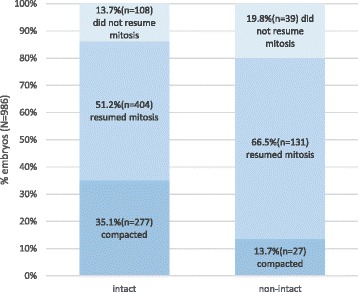
Proportion of intact and non-intact that resumed mitosis or compacted after overnight culture. Legend: There is a lower proportion of cleaving embryos and higher proportion of compacted embryos in intact versus non-intact embryos (Cochran-Armitage test *p*-value < 0.0001)

From the total number of embryos included in this analysis (*n* = 986), 80 % (*n* = 789) survived with all blastomeres intact and 20 % (*n* = 197) survived but the embryo was not intact after survival (non-intact). The proportion of embryos that resumed mitosis, that did not resume mitosis and that compacted after overnight culture is shown in Fig. 1 separately for intact and non-intact embryos. The mitosis resumption rate was significantly higher for intact embryos than for non-intact embryos (86 vs. 70 %, Chi-square *p*-value = 0.001).

**Fig. 2:**
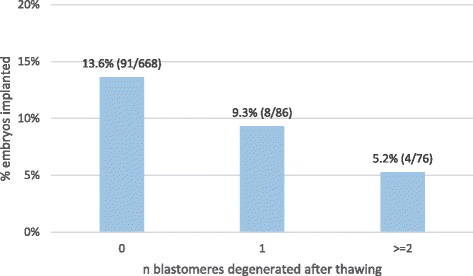
IR per embryo transferred in relation to the number of blastomeres degenerated after warming. Legend: Implantation rate (IR) (n embryos implanted/n embryos transferred) was calculated for embryos transferred in SET or DET with 0 % or 100 % implantation. Cochrane Armitage *p*-value = 0.02

**Fig. 3:**
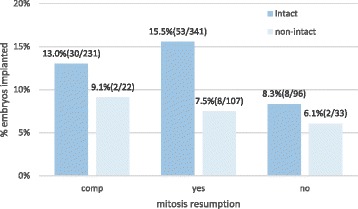
IR per embryo transferred in relation to intact survival and mitosis resumption. Legend: Implantation rate (IR) (n embryos implanted/n embryos transferred) was calculated for embryos transferred in SET or DET with 0 % or 100 % implantation. There is significantly higher implantation rate in intact embryos than in non-intact embryos with mitosis resumption (fisher’s exact *p*-value = 0.03), but no difference between both groups for embryos without mitosis resumption or compacted embryos

The 986 embryos were transferred in 671 FET cycles and resulted in an overall IR per transferred embryo of 16.9 % (167/986). For the subset of 830 out of 986 embryos with known implantation (i.e., transferred in SET or DET with 0 or 100 % implantation), the IR per embryo transferred was 12.4 % (103/830). When looking at the IR in relation to the number of cells lost, a significant decrease in IR was observed with the loss of 1 or ≥2 cells (Fig. 2) (Cochran-Armitage *p*-value = 0.02). When comparing IR between intact and non-intact embryos in relation to mitosis resumption (Fig. 3), we observed that within embryos that resumed mitosis, intact embryos had significantly higher IR than non-intact embryos (15.5 vs. 7.5 %, Fisher’s exact *p*-value = 0.03). Contrarily, within embryos that compacted and within embryos that did not resume mitosis, intact and non-intact embryos had comparable IRs (13.0 vs. 9.1 %, Fisher’s exact *p*-value > 0.99; 8.3 vs. 6.1 %, Fisher’s exact *p*-value > 0.99) (Fig. 3).

**Fig. 4:**
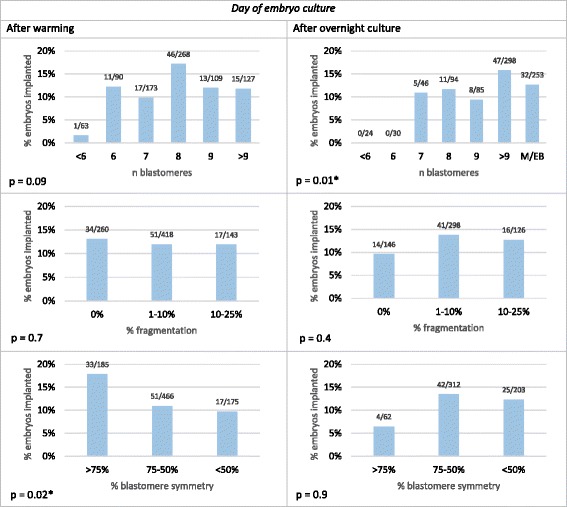
IR per embryo transferred in relation to number of blastomeres, fragmentation and symmetry, evaluated manually. Legend: Implantation rate (IR) (n embryos implanted/n embryos transferred) was calculated for embryos transferred in SET or DET with 0 or 100 % implantation. *P* values were calculated using Cochrane-Armitage test. Significant *p*-values (<0.05) are marked with *. Higher number of blastomeres after overnight culture and higher blastomere symmetry after warming were significantly associated with higher IR. Embryos in morula stage after warming (*n* = 4) are included in the group of >9 blastomeres. Fragmentation and symmetry was not evaluated for embryos in M or EB stage (*n* = 4 after warming; *n* = 253 after overnight culture). Embryos with >25 % fragmentation after warming are not included in the graphs because of the low number (*n* = 5 after warming, *n* = 7 after overnight culture). M = morula; EB = early blastocyst

In Fig. 4, the IR per embryo transferred is shown in relation to the number of blastomeres, the degree of fragmentation and the degree of symmetry, which were evaluated manually on the day of warming and after overnight culture. Cochrane-Armitage test supported a significant relation between IR and number of blastomeres after overnight culture (*p* = 0.01) but not with number of blastomeres after thawing (*p* = 0.08). In contrast, for the degree of symmetry, statistical support was obtained for a relation with IR when it was evaluated after warming (*p* = 0.01) but not after overnight culture (*p* = 0.9). For fragmentation, no statistical evidence was obtained for its relation with IR neither when evaluated after warming (*p* = 0.7) nor after overnight culture (*p* = 0.4).

### Morphometric characteristics

From the 986 embryos included, 654 embryos were analyzed for morphometrics using the computer assisted analysis (Table 2). Due to missing images (*n* = 59 on day 1, *n* = 21 on day 3, *n* = 41 after warming) and/or compaction (*n* = 36 on day 3, *n* = 20 after warming, *n* = 405 after overnight culture), morphometric analysis could not be performed resulting in missing values. Taking this into account, total cell volume was measured on 595 embryos on day 1, on 597 embryos at freezing (day 3), on 593 embryos after warming and on 222 embryos after overnight culture. Fragmentation was calculated at freezing (day 3) on 547 embryos (4 embryos were excluded due to fragmentation ≤ -20 %).

**Table 2:** Morphometric characteristics of vitrified/warmed embryos measured at each evaluation moment

		Day 1	At freezing (Day 3)	After warming	After overnight culture
Total cell volume (μm^3^)	n	595^a^	597^b^	593^c^	222^d^
	Mean ± SD	827,074 ± 85,461	724,173 ± 88,648	693,552 ± 117,108	626,881 ± 122,672
Fragmentation• (%)	n	-	547^a,b,e^	-	-
	Mean ± SD	-	12.9 ± 9.0	-	-
Volume change (%)••	n	-	-	560^b,c,f^	216^c,d,g^
	Mean ± SD	-	-	−4.0 ± 14.5	−6.9 ± 11.3
Blastomere symmetry (%)•••	n	-	597^b^	593^c^	222^d^
	Mean ± SD	-	73.7 ± 8.5	73.4 ± 9.3	68.3 ± 8.8

For each characteristic the number of embryos from which the characteristics were measured and the mean value ± standard deviation is shown

All morphometric characteristics were calculated based on the total cell volume (TCV) of the embryo at the particular evaluation moment

•Difference of TCV in percentage between embryo at freezing and same embryo on day 1. This measurement assumes that embryo TCV stays constant through the development. Due to the process of vitrification we cannot assume this, thus fragmentation was not calculated after warming

••Difference of TCV in percentage between the same embryos at two consecutive evaluation moments

•••Difference of diameter in percentage between the biggest and the smallest blastomere of the same embryo at the same evaluation moment

^a^59/654 embryos had no picture on Day1

^b^21/654 embryos had no picture at freezing and 36/654 embryos were compacted at freezing

^c^41/654 embryos had no picture after warming and 20/654 embryo were compacted after warming

^d^27/654 embryos had no picture after overnight culture and 405/654 embryos were compacted after overnight culture

^e^9/59 embryos with no picture on Day 1 had also no picture at freezing and 4/59 embryos with no picture on Day 1 were compacted at freezing. 4/654 embryos had fragmentation ≤ -20 % and were excluded

^f^3/41 embryos with no picture after warming were compacted before freezing, 6/41 embryos with no picture after warming had no picture at freezing. 15/20 embryos that were compacted after warming were also compacted before freezing

^g^1/27 embryos with no picture after overnight culture was compacted after warming. 21/27 embryos with no picture after overnight culture had no picture after warming. 14/405 embryos compacted after overnight culture had no picture after warming. 19/405 embryos compacted after overnight culture were compacted after warming

From a descriptive point of view, morphometrics revealed a decrease in TCV over time, both after warming and after overnight culture (Table 2). Specifically, embryos lost in average 4 % of their volume after warming, when compared to their volume before vitrification, and they lost an additional 7 % after overnight culture, when compared to their volume after warming. Moreover, we observed no change in blastomere symmetry after warming i.e., an average of 74 % symmetry was observed both before and after warming. After overnight culture, symmetry slightly decreased to 68 %.

From the subset of 654 embryos that were analyzed for morphometrics, 544 were transferred in SET or DET with 0 or 100 % implantation and therefore used in relating morphometric characteristics with IR. In Table 3, we show the IR in relation to TCV, fragmentation, volume change and symmetry. Despite our selection criteria for vitrification based on manual embryo morphological scoring (≥6 cells, ≤ 25 % fragmentation and >50 % symmetry on day 3), 48 embryos showed >25 % fragmentation before vitrification when measured with morphometrics. Nevertheless, IR was neither related to fragmentation before vitrification (Cochrane-Armitage test *p*-value = 0.6), nor to volume change after warming (Chi-square *p*-value > 0.9), nor to volume change after overnight culture (Fisher’s exact *p*-value = 0.3) nor to symmetry at freezing (Chi-square *p*-value = 0.1), after warming (Chi-square *p*-value = 0.3) or after overnight culture (Fisher’s exact *p*-value = 0.3).

**Table 3:** Implantation rate according to embryo morphometric characteristics

	Category	At freezing (Day 3)	After warming	After overnight culture
Fragmentation	0 %	12.0 % (6/50)	-	-
	1–10 %	13.0 % (17/131)	-	-
	10–25 %	8.0 % (18/224)	-	-
	>25 %	14.6 % (7/48)	-	-
	Missing	91	-	-
	*p*-value	0.6^a^		
Volume change (only intact embryos)	Gain	-	11.6 % (20/172)	5.1 % (2/39)
	Loss	-	11.1 % (24/216)	11.3 % (12/106)
	Missing	-	71	306
	*p*-value		>0.99^b^	0.3^c^
Symmetry	>75 %	13.7 % (29/211)	13.5 % (30/222)	2.7 % (1/37)
	75–50 %	8.9 % (25/281)	10.2 % (27/266)	9.7 % (14/145)
	<50 %	3	3	2
	Missing	49	51	360
	*p*-value	0.1^b^	0.3^b^	0.3^c^

Implantation rate (n embryos implanted/n embryos transferred) was calculated for embryos transferred in SET or DET with 0 or 100 % implantation

^a^Cochrane-Armitage trend test *p*-value

^b^Chi-square *p*-value

^c^Fisher’s exact *p*-value

## Discussion

This study aimed to find new parameters to improve the selection criteria both for vitrifying and for transferring day 3 embryos after warming. For this purpose, we investigated the IR of day 3 vitrified/warmed embryos in relation to standard morphological characteristics (intact survival, mitosis resumption, number of blastomeres, symmetry and fragmentation) as well as to morphometric characteristics (fragmentation, symmetry and volume change), both after warming and after overnight culture. Due to the fact that embryo scoring has an important subjective component we included, for the first time, in day 3 embryos after warming, a computer based morphometric analysis of symmetry, fragmentation and volume change, due to their reduced subjectivity and intra-observer variability [28]. In contrast with our hypothesis, implantation rate after warming was not related to morphometric evaluation of symmetry, fragmentation and volume change, and was only determined by morphological characteristics. We found that 80 % of the embryos survived intact after vitrification/warming and 67 % of embryos contained >9 cells after overnight culture. In addition, they did not experience significant changes in symmetry, fragmentation and volume. We observed that when mitosis resumption was present, intact embryos had a significantly higher IR than non-intact embryos. Remarkably, embryos with <6 cells after warming or with ≤6 cells after overnight culture had extremely low IR (<1 %).

For slow frozen/thawed embryos, the relation of intact survival and mitosis resumption with IR has been widely described [6–9]. However, to the best of our knowledge, at the moment only one study shows a relation of intact survival and mitosis resumption with IR in vitrified embryos [10]. In slow frozen embryos, the presence of mitosis resumption improved IR [8–10] while IR decreased with the number of cells lost [8]. More concretely, the loss of >25 % of the embryo after thawing was detrimental for IR [6] and specifically for 7- and 8-cell embryos the loss of >2 cells reduced significantly IR [7]. In the case of vitrified embryos, Van Landuyt et al. [10] found no effect on IR of 294 SET when embryos lost up to 2 cells and as long as the embryo continued to cleave after overnight culture. Our results in 830 vitrified embryos do not support these findings, since we observed that non-intact embryos had lower IR compared to intact embryos even if mitosis resumption was present (7.5 vs. 15.5 %, *p* = 0.03). To the best of our knowledge, we are the first group to report that the number of cells damaged had no effect on IR if the embryo had compacted after overnight culture. In addition, if mitosis resumption was not present, intact and non-intact embryos reached the same IR. Besides intact survival and mitosis resumption, the specific number of blastomeres after overnight culture is a characteristic that has been described to influence the IR of survived day 2 embryos after slow freezing [36, 37]. To the best of our knowledge, our study describes for the first time the IR in relation to the specific number of blastomeres in vitrified/warmed embryos. According to our results, the number of blastomeres after overnight culture has an effect on implantation regardless of cell stage after warming (*p* = 0.01). The majority of the warmed and survived embryos had 8 cells after warming (32.0 %) and >9 cells after overnight culture (66.9 %), which is comparable with results reported by Van Landuyt et al. [10]. We observed that embryos with 8 cells after warming or >9 cells after overnight culture –including compacted embryos– reached the highest IR (>15 %). The IR of embryos in other cell stages was at least 5 % lower (Fig. 4). Interestingly, embryos with <6 cells after warming or with ≤6 cells after overnight culture had an IR <1 %.

In the cryopreservation of cleavage stage embryos, intact survival, mitosis resumption and IR are the key performance indicators defined by the Alpha Scientists in Reproductive Medicine [11]. This group of experts defined competence level and benchmark for each of these parameters. Our intact survival rate (80 %) is between the competence level (70 %) and the benchmark (85 %) and is in line with previous publications, which obtained 75–83 % intact survival rate after warming [4, 10, 19–21, 24, 26, 38]. In the case of the mitosis resumption rate, the Alpha Scientists in Reproductive Medicine [11] defined this benchmark only for intact embryos, which should be a maximum of 10 % less than the good quality fresh embryos at the same stage. However, since in our lab the embryos are transferred on day 3, no data in fresh embryos were available to compare the cleavage to day 4 of vitrified/warmed embryos. Nevertheless, the literature describes similar mitosis resumption rates (78–88 %) to our results (85 %) in embryos survived after vitrification [4, 10, 26]. Hence, other studies also showed that mitosis resumption is higher in intact than in non-intact embryos (93 vs. 70 %, [10] and 81 vs. 69 % [4]) supporting our results (86 vs. 70 %; *p* = 0.001). Furthermore, we observed that the % of embryos in the stage of morula or early blastocyst increased from 0.4 % after warming to 31 % after overnight culture, indicating a good overnight development.

The overall IR per transferred embryo in our study is 16.9 % (167/986), which in line with previous studies (16–21 % [4, 26]). Since our aim was to relate individual embryo morphological characteristics with IR we needed to calculate the IR per embryo transferred using only embryos transferred in SET or DET with either 0 or 100 % implantation. Thus, IR was known for a subset of 830/986 embryo and resulted in 12.4 % (103/830). The decrease of IR per embryo transferred in respect to the overall IR is explained by the exclusion of DETs with implantation of only one embryo. The benchmark described for IR of cleavage stage embryos after vitrification in the Alpha consensus meeting on cryopreservation [11] is the same as for mitosis resumption, i.e. maximum of 10 % less than the fresh embryos of the same stage, and such population is not available for comparison in our lab. In summary, our analysis of morphological characteristics is in line with previous studies and can reach the benchmark established by experts.

Despite the fact that morphometric characteristics were not related to IR in day 3 vitrified/warmed embryos, the measurement of these characteristics revealed several new descriptive features regarding fragmentation, blastomere symmetry and volume change that could not be observed otherwise (i.e. with manual evaluation). In respect to fragmentation, an average of 13 % fragmentation was observed before vitrification. Even though embryos selected for cryopreservation can have up to 25 % fragmentation, the majority of embryos presented 1–10 % fragmentation, both after warming and after overnight culture (50 and 36 % respectively). This might indicate a subjective preferential selection of embryos with ≤10 % fragmentation for cryopreservation by the observers. If this is true, the use of morphometrics to select embryos for cryopreservation with <25 % fragmentation would lead to an increase of the number of embryos with >10 % fragmentation cryopreserved. If contrarily, there is no preferential selection by the observers, the lower proportion of embryos with >10 % fragmentation could be explained by the association of other suboptimal characteristics to the degree of fragmentation. In other words, embryos with >10 % fragmentation might have as well an insufficient number of blastomeres or % symmetry that do not fulfil the criteria for cryopreservation. Further studies about manual and computer assisted intra- and inter-observer variability in selecting embryo for cryopreservation as well as studies about association of % fragmentation with % symmetry and number of blastomeres would be needed to support these hypothesis. As for blastomere symmetry, when measured using morphometrics, the average symmetry was similar before vitrification and immediately after warming (73.7 vs. 73.4 %) but it experienced a slight decrease of 5 % after overnight culture. The decrease of symmetry after overnight culture could also be seen with the manual evaluation of symmetry (Table 1). In that case, we found higher IR with a higher blastomere symmetry only at the day of warming. Nevertheless, we would rather carefully interpret this result since this relation was not significant when symmetry was evaluated after overnight culture and was not significant when symmetry was evaluated using morphometrics. The only results available regarding degree of symmetry in cryopreserved embryos describe a positive influence of high blastomere symmetry on day 2–3 during fresh culture on implantation after thawing [8]. The putative importance of the blastomere symmetry on day 3 could be explained by the fact that equally sized blastomeres are expected when the embryo has 4, 8 or 16 cells. On day 3, 8-cell stage embryos are the majority, both before vitrification and after warming, while, on day 4 (after overnight culture), the majority of the embryos have >9 cells but very few have 16 cells due to appearance of compaction, thus presenting lower blastomere symmetry. In regard to the volume change, the embryos lost a mean of 4 % of the volume after warming and 7 % of the volume after overnight culture, nevertheless with a high variance (Table 2), indicating that some embryos do experience a volume increase in spite of the vitrification/warming procedure. This variability in volume caused by vitrification has been described in oocytes [11, 39, 40] but has never been quantified or related to clinical outcome. We hypothesized that volume change caused by vitrification might be an indicator of implantation potential. Our results show that although the majority of embryos have a reduced volume after warming compared to before vitrification, some embryos do recover their volume or even increase it. However, embryos with gain or with loss of volume did not have different IR suggesting that the volume change does not influence the embryo developmental and implantation potential. It is important to notice that morphometric parameters cannot be measured in compacted embryos, therefore, the number of missing values in the evaluation of morphometric characteristics after overnight culture need to be interpreted with caution. Nevertheless, the fact that vitrified embryos present a high compaction rate after overnight culture is already a good prognosis indicator, which decreases the importance of morphometric measurements at that stage.

In our opinion, our criteria to select day 3 embryos for cryopreservation using vitrification is strict enough, even though more strict criteria are proposed by the Alpha consensus [11]. While we select supernumerary embryos for freezing if they have ≥ 6 cell stage on day 3, ≤ 25 % fragmentation and >50 % symmetry on day 3, Alpha consensus only recommends freezing embryos that had 4 cells on day 2 and 8 cells on day 3 –excluding all embryos that had a different evolution and cell number–, <10 % fragmentation, stage-specific cell size –giving no specification on the symmetry– and no multinucleation. We observed no effect of blastomere symmetry and fragmentation on implantation rate, which suggests that embryos with reduced IR due to these characteristics were already excluded before cryopreservation. Therefore, excluding vitrified and warmed embryos for transfer based on fragmentation >10 % and blastomere symmetry <25 % would not improve IR, but would in contrast lead to discarding embryos with acceptable implantation potential. The most important parameter to take into account in the evaluation of vitrified day 3 embryos after warming according to our study is the number of blastomeres, since this is an indicator of both intact survival and mitosis resumption. According to the IR observed in respect to the number of blastomeres after warming, we would strongly recommend to (1) preferentially warm the 8 cell embryos, because they have the highest IR and (2) to warm an extra embryo if available when an embryo does not survive intact with <6 cells, due to their extremely low IR, as recommended before [10]. We agree with the recommendation [10] that, in case of 8-cell embryos, when more than 2 cells are damaged an extra embryo should be thawed and in case of 6- and 7-cell embryos, when more than one cell is damaged another embryo should be thawed.

## Conclusions

In conclusion, after warming and survival, IR of vitrified embryos is determined by the number of cells lost, by the occurrence of mitosis resumption, and by the specific number of blastomeres present. This IR was not affected by fragmentation, blastomere symmetry and volume change, most likely because embryo selection for these criteria takes place before vitrification.

## Abbreviations

ART, assisted reproductive technology; DET, double embryo transfer; FET, frozen-thawed embryo transfer; IR, implantation rate; IR, implantation rate; IVF, in vitro fertilization; OR, oocyte retrieval; SET, single embryo transfer; TCV, total cell volume
